# Bayesian Inference of Synaptic Quantal Parameters from Correlated Vesicle Release

**DOI:** 10.3389/fncom.2016.00116

**Published:** 2016-11-25

**Authors:** Alex D. Bird, Mark J. Wall, Magnus J. E. Richardson

**Affiliations:** ^1^Theoretical Neuroscience Group, Warwick Systems Biology Centre, University of WarwickCoventry, UK; ^2^Ernst Strüngmann Institute for Neuroscience, Max Planck SocietyFrankfurt, Germany; ^3^Frankfurt Institute for Advanced StudiesFrankfurt, Germany; ^4^School of Life Sciences, University of WarwickCoventry, UK; ^5^Warwick Mathematics Institute, University of WarwickCoventry, UK

**Keywords:** correlation, Bayesian, EPSP, synapse, quantal, stochastic, plasticity

## Abstract

Synaptic transmission is both history-dependent and stochastic, resulting in varying responses to presentations of the same presynaptic stimulus. This complicates attempts to infer synaptic parameters and has led to the proposal of a number of different strategies for their quantification. Recently Bayesian approaches have been applied to make more efficient use of the data collected in paired intracellular recordings. Methods have been developed that either provide a complete model of the distribution of amplitudes for isolated responses or approximate the amplitude distributions of a train of post-synaptic potentials, with correct short-term synaptic dynamics but neglecting correlations. In both cases the methods provided significantly improved inference of model parameters as compared to existing mean-variance fitting approaches. However, for synapses with high release probability, low vesicle number or relatively low restock rate and for data in which only one or few repeats of the same pattern are available, correlations between serial events can allow for the extraction of significantly more information from experiment: a more complete Bayesian approach would take this into account also. This has not been possible previously because of the technical difficulty in calculating the likelihood of amplitudes seen in correlated post-synaptic potential trains; however, recent theoretical advances have now rendered the likelihood calculation tractable for a broad class of synaptic dynamics models. Here we present a compact mathematical form for the likelihood in terms of a matrix product and demonstrate how marginals of the posterior provide information on covariance of parameter distributions. The associated computer code for Bayesian parameter inference for a variety of models of synaptic dynamics is provided in the Supplementary Material allowing for quantal and dynamical parameters to be readily inferred from experimental data sets.

## 1. Introduction

The statistics and dynamics of stochastic synaptic filtering determine how information is communicated between neurons. Synapses act as activity-dependent filters on the transfer of neuronal signals, suppressing or amplifying trains of inputs to the postsynaptic cell relative to isolated stimuli, in a phenomenon known as short-term plasticity or synaptic dynamics (Zucker and Regehr, 2002; Abbott and Regehr, 2004; Mongillo et al., 2008). An action potential in the presynaptic cell triggers an influx of Ca^2+^ into synaptic terminals, causing a probabilistic all-or-none release of neurotransmitter at each active vesicle docking site on the presynaptic membrane. The neurotransmitter binds to channels on the postsynaptic cell resulting in, for example, an excitatory post-synaptic potential (EPSP) “built up statistically of the all-or-none events that are similar in size and distribution to spontaneous miniature” postsynaptic potentials (del Castillo and Katz, 1954). Depletion of vesicles available at active sites can cause an activity-dependent reduction in synaptic efficacy (Eccles et al., 1941) whereas a build-up of Ca^2+^ in the presynaptic terminal can increase the probability of neurotransmitter release (Dudel and Kuffler, 1961). Synaptic transmission is thus both fundamentally stochastic (del Castillo and Katz, 1954; Fatt and Katz, 1952; Stein, 1965) and history dependent (Furukawa et al., 1982; Abbott, 1997; Tsodyks and Markram, 1997).

Initial analyses of paired-cell data used the amplitude distribution of isolated EPSPs to identify quantal peaks corresponding to sums of similar mini amplitudes (Boyd and Martin, 1956; Liley, 1956; Kuno, 1964; Kuno and Weakly, 1972; Bennett and Florin, 1974; Bekkers, 1994); for a review see Bennett and Kearns (2000). While this was an effective approach for extracting the properties of neuromuscular synapses (del Castillo and Katz, 1954) the greater variation in mini amplitudes at central synapses (Hanse and Gustafsson, 2001; Franks et al., 2003; Hardingham et al., 2010) necessitated different techniques to recover robust results in the central nervous system. Mean-variance analysis was developed to obtain estimates of the maximum number of vesicles that can be released by a single stimulus (Silver et al., 1998; Clements, 2003; Silver, 2003). Initial applications relied on conducting experiments under a variety of conditions, in particular varying the extracellular Ca^2+^ concentration to alter the vesicle release probability (Foster and Regehr, 2004; Birò et al., 2005). Brémaud et al. (2007) and Loebel et al. (2009) increased the practicality of the method by using short-term vesicle depletion to vary the effective release probability under a single experimental condition. Their analyses showed that multiquantal release underlies the wide range of EPSP amplitudes observed (Song et al., 2005; Lefort et al., 2009) and that, in general, it is not the case that the number of distinct anatomical contacts equals the maximum number of readily-releasable vesicles as was put forward by the *single-vesicle hypothesis* (Kuno, 1971; Korn et al., 1981).

More recent approaches have introduced a principled Bayesian approach to infer synaptic parameters. Bayesian inference determines the extent to which experimental evidence supports a given set of model parameters. This relies on the fact that the probability of a certain model being correct given observed data is proportional to the probability of observing that data given that the model is correct. As such it makes maximal use of data, including every observation rather than extracting moments as in previous approaches. This framework was first applied to neurophysiological synaptic data by Turner and West (1993) to extract the number of components in a unitary EPSP. More recently, McGuinness et al. (2010) used Bayesian analysis to measure presynaptic Ca^2+^ concentrations and Bhumbra and Beato (2013) used an exact Bayesian approach to extract quantal parameters from measurements of isolated EPSPs, demonstrating that accurate parameter estimates could be obtained from less data than with existing mean-variance methods.

Inference on isolated EPSPs, however, does not allow recovery of synaptic parameters associated with short-term plasticity. Costa et al. (2013) addressed this issue in a Bayesian framework using the Tsodyks-Markram model of short-term plasticity (Tsodyks et al., 1998) with a likelihood that approximated synaptic amplitude distributions during patterned input as uncorrelated Gaussians around the mean amplitudes. Though this approach does not account for correlations between closely-timed synaptic events, the method nevertheless allowed for accurate inference of a number of synaptic parameters. However, correlations between successive PSPs, which can be significant even at stimulation rates below 10 Hz, (del Castillo and Katz, 1954; Thomson et al., 1993; Fuhrmann et al., 2002) can provide a useful source of additional information for inferring model parameters. This is particularly the case for data sets that feature only a few repreated stimulations or only one series of patterned PSPs such as would be the case for spontaneous *in-vivo* recordings.

The main barrier to extending the Bayesian approach to a model that allows simultaneous recovery of both quantal and dynamic properties is the calculation of the likelihood of seeing a particular train of amplitudes in response to a certain pattern of presynaptic stimuli. This probability is dependent on the correlated vesicle releases during previous events and the number of possibilities therefore grows exponentially with the number of PSPs. Naively, this would appear to make the problem intractable. However, two independent studies (Barri et al., 2016; Bird, 2016) recently provided a solution to this problem by exploiting the underlying Markovian nature of the problem thereby allowing for the computation of the exact probability of a given set of observed amplitudes with a complexity that grows only linearly with PSP number. Here we develop the method, originally presented in Bird (2016), to show how the likelihood may be written in a compact mathematical form as a matrix product. This allows for efficient calculation of the posterior distribution from which, for example, the covariance of the inferred parameters can be analyzed. Our complete Bayesian method may be thought of as combining the method for inferring quantal parameters for isolated PSPs developed by Bhumbra and Beato (2013) with the method for inferring mean synaptic dynamics (without including correlations) developed by Costa et al. (2013). As well as describing the mathematical solution we additionally provide the software code to perform Bayesian inference for a variety of models of synaptic dynamics as part of this publication.

## 2. Methods

In this section we define the general class of synaptic models our inference procedure applies to before specifying a commonly used depression-facilitation model of neurotransmitter release that will be used for illustrative purposes. The coupling of the presynaptic model to the post-synaptic voltage response is then defined.

### 2.1. The class of synaptic dynamics models

The method presented here is applicable to a broad class of synaptic models. The synapses this method can be applied to are assumed to have a number *n* of vesicle release sites to which neurotransmitter vesicles can dock. On arrival of the *m*th presynaptic spike at time *t*_*m*_ neurotransmitter is released independently from each docked vesicle with probability *u*_*m*_. The binary occupancy variable *x*(*t*) for single release site obeys

(1) $$\begin{array}{l} \frac{dx}{dt}=\left(1-x\right)\underset{\left\{t_{r}\right\}}{\sum }\delta \left(t-t_{r}\right)-x\underset{\left\{t_{m}\right\}}{\sum }\delta_{m}\delta \left(t-t_{m}\right) \end{array}$$

where *t*_*r*_ are restock events (which occur at a rate that may be dependent on the presynaptic action potential times) and δ_*m*_ is a binary random variable signifying release of neurotransmitter that is equal to 1 with probability *u*_*m*_ and 0 otherwise. The stochasticity in *t*_*r*_ and δ_*m*_ is considered to be statistically independent across the *n* vesicle release sites. Note also that in this formulation any dynamic quantity (such as *x*(*t*)) multiplying a Dirac-delta function is evaluated just before the arrival of the impulse. The expected change in occupancy before and after a presynaptic action potential can be straightforwardly derived to give

(2) $$\begin{array}{l} {\langle x\rangle }_{m}^{⊕}={\langle x\rangle }_{m}^{⊖}-u_{m}{\langle x\rangle }_{m}^{⊖} \end{array}$$

where ${\langle x\rangle }_{m}^{⊖}$ is the probability that a release site is occupied just before and ${\langle x\rangle }_{m}^{⊕}$ just after the *m*th spike. Similarly, the probability of occupancy just before (*m* + 1)th AP can be related to the occupancy just after the *m*th AP as

(3) $$\begin{array}{l} {\langle x\rangle }_{m+1}^{⊖}=1-\left(1-{\langle x\rangle }_{m}^{⊕}\right)\left(1-g_{m}\right) \end{array}$$

where *g*_*m*_ is the restock probability. For certain models *g*_*m*_ can depend on the history of the presynaptic APs. Together the recursion relations (3) and (5) give the occupancy probability for an arbitrary train of presynaptic action potentials. The initial condition is typically taken as being ${\langle x\rangle }_{1}^{⊖}=1$, where all release sites are stocked. These dynamics cover a range of models such as vesicle depression (Tsodyks and Markram, 1997), depression with facilitation (Varela et al., 1997; Tsodyks et al., 1998; Fuhrmann et al., 2002), frequency-dependent recovery (Fuhrmann et al., 2004) and augmented recovery (Wang and Kaczmarek, 1998; Hosoi et al., 2007). For an in-depth discussion, see Appendix A.

### 2.2. Illustrative synaptic model with depression and facilitation

To provide an example of the method we use a commonly used model that combines a depression mechanism caused by vesicle release and a constant restock rate with a facilitation mechanism that models the effect of increased release probability due to transient increases in calcium concentrations in the presynaptic terminal (Varela et al., 1997; Tsodyks et al., 1998; Fuhrmann et al., 2002). The restock process is Poissonian and has constant rate 1/τ_*D*_, where τ_*D*_ is commonly referred to as the depression time constant; therefore the restock probability required for Equation (3) is simply

(4) $$\begin{array}{l} g_{m}=1-e^{-T_{m}/\tau_{D}} \end{array}$$

where *T*_*m*_ = *t*_*m*+1_ − *t*_*m*_ is the time between the *m*th and (*m* + 1)th APs. Let *p*_0_ be the baseline value of the probability of release, and *p*_1_ be the facilitated release probability immediately after an isolated spike. Let *u*(*t*) be the time-dependent release probability. In the absence of stimulus, *u*(*t*) decays back to *p*_0_ with timescale τ_*F*_. The dynamics of *u*(*t*) therefore obeys

(5) $$\begin{array}{l} \frac{du}{dt}=\frac{1}{\tau_{F}}\left(p_{0}-u\right)+\left(1-u\right)\left(\frac{p_{1}-p_{0}}{1-p_{0}}\right)\underset{t_{m}}{\sum }\delta \left(t-t_{m}\right) \end{array}$$

where the (1 − *u*) prefactor of the Delta functions prevents the probability going above unity. In this setup *u* = *p*_0_ if the previous spike was a long time ago, then on the arrival of a spike it jumps to *u* = *p*_1_. Because it is a facilitation model we have *p*_0_ < *p*_1_ < 1. Note that this formulation of parameters allows the facilitated release probability *p*_1_ to be fixed independently of the initial release probability *p*_0_ and maps directly to the original quantal facilitating and depressing synaptic model of Fuhrmann et al. (2002) with *p*_0_ = *U*_SE_ and *p*_1_ = *U*_SE_ + (1 − *U*_*SE*_)*U*_1_ using that paper's notation. The values of *u*(*t*) just after the *m*th and before the (*m* + 1)th action potentials ($u_{m}^{⊕}$ and *u*_*m*+1_ respectively) are defined by the following recursion relations

(6) $$\begin{array}{l} u_{m}^{⊕}=u_{m}+\left(1-u_{m}\right)\;(\frac{p_{1}-p_{0}}{1-p_{0}})\text{ and}\\ u_{m+1}=p_{0}+\left(u_{m}^{⊕}-p_{0}\right)e^{-T_{m}/\tau_{F}} \end{array}$$

where the initial conditions are that *u*_1_ = *p*_0_ and $u_{1}^{⊕}=p_{1}$. This gives the release probability before each presynaptic spike required for Equation (2). The dynamics of the restock probability *g* are unaffected and are given by Equation (4). A special case of this model that has one less free parameter is when the release probability doubles after an isolated spike and so *p*_1_ = 2*p*_0_ (Tsodyks et al., 1998).

### 2.3. EPSP amplitude distribution

The post-synaptic amplitude statistics for single vesicle release of neurotransmitter is modeled by a gamma distribution with mean μ_*a*_ and standard deviation σ_*a*_ (μ_*a*_ is assumed to be greater than σ_*a*_ to ensure that a zero amplitude is not the most likely). This is preferred over a normal distribution on empirical grounds and ensures that amplitudes are always positive (Robinson, 1976; Hanse and Gustafsson, 2001; Bhumbra and Beato, 2013). However, it is reasonable to assume that background noise is normal with standard deviation σ_*b*_ and is independent of EPSP amplitude. Note that this choice of amplitude generation is identical to that described for isolated EPSPs in (Bhumbra and Beato, 2013). With this choice, if *k* vesicles release neurotransmitter from among the *n* possible release sites, the observed EPSP amplitude *A* is written *A* = ψ + ϕ where ψ is the release-dependent component and ϕ the independent Gaussian noise. Because ψ is the sum of *k* individual quantal amplitudes, each of which are independently identically gamma distributed, its distribution is also gamma-distributed with

(7) $$\begin{array}{lll} \text{P}\left[\psi \right]=\frac{\lambda^{\beta }}{\Gamma \left(\beta \right)}e^{-\lambda \psi }\psi^{\beta -1} & \text{where} & \beta =k\frac{\mu_{a}^{2}}{\sigma_{a}^{2}}\text{ and }\lambda =\frac{\mu_{a}}{\sigma_{a}^{2}}. \end{array}$$

The distribution for the measured EPSP amplitude *A*, given *k* release events, is therefore a convolution between the gamma and normally distributed components of the noise

(8) $$\begin{array}{l} \text{P}\left[A|k\right]=\frac{\lambda^{\beta }}{\Gamma \left(\beta \right)}\frac{1}{{\left(2\pi \sigma_{b}^{2}\right)}^{\frac{1}{2}}}\int_{0}^{\infty }dye^{-\lambda y}y^{\beta -1}e^{-\frac{{\left(A-y\right)}^{2}}{2\sigma_{b}^{2}}}. \end{array}$$

An approach for numerically calculating this integral efficiently is provided in Appendix B.

### 2.4. Computational methods and code

An exhaustive grid-based derivation of the likelihood function for the depression-only model (see Appendix) is just within the capabilities of easily accessible computers at the time of writing. However, for more involved models with a greater number of parameters this becomes impracticable and a Markov Chain Monte Carlo (MCMC) approach was used instead. Here priors are taken to be flat (uninformative) for all parameters for illustrative reasons: more informative priors can be included as required. For the MCMC implementation, parameter space is discretised into a grid and the sampler is initialized at a random point consistent with any restrictions on the model parameters. Moves are proposed to each adjacent grid point with equal probability and accepted or rejected based on the log-likelihood ratio of the current and proposed points. Convergence of the sampler was examined by comparing the distributions resulting from chains initiated in different locations. It is straightforward to extend this transparent implementation in our code to include more sophisticated methods such as slice sampling. We provide MATLAB and JULIA code for the Bayesian inference of synaptic parameters from measurements of synaptic amplitudes using the Metropolis-Hastings sampling method (Metropolis et al., 1953; Hastings, 1970) described above as part of the Supplementary Material. The code covers the major synaptic dynamics models including: depression only, depression-facilitation, release-independent depression and frequency-dependent recovery. The models are described in the Appendix.

### 2.5. Synthetic and experimental data

To test the model we used both artificial and experimental data sets. Synthetic data with known parameters was generated from the synaptic-dynamics models and consisted of a series of stimulation times and stochastically determined EPSP amplitudes. For experimental data sets the data analyzed consisted of EPSP amplitudes combined with their arrival times. The data, comprising paired whole-cell patch-clamp recordings of layer-5 pyramidal neurons, was taken from a previous study (Kerr et al., 2013). Here data obtained in control conditions and in the presence of 100μM bath-applied adenosine was used. Presynaptic cells were stimulated with square-pulse currents of 5ms duration and magnitude sufficient to reliably induce a single action potential without causing bursting. Stimulation consisted of 10 spikes at 20 − 50 Hz with 10 s between traces ensuring sufficient time for full recovery and statistical independence for the next sweep. For each presentation of the same presynaptic stimulus the amplitudes of the individual EPSPs were extracted from the postsynaptic voltage trace using the voltage deconvolution method (Richardson and Silberberg, 2008) providing a vector of 10 EPSP amplitudes.

## 3. Results

In this section we first summarize the broad class of synaptic models our methodology applies to. We then describe the nature of the computational problem involved in calculating exact correlated likelihoods. We go on to show how the probability of observing a set of numbered release events for a chain of presynaptic action potentials can be calculated using a Markovian property. By coupling this result to the miniature PSP distribution, the full likelihood for an observed PSP amplitude train is then derived in the form of a matrix multiplication. Finally, we demonstrate the method on both synthetic and experimental data, recovering the shift in synaptic dynamics caused by the neuromodulator adenosine.

### 3.1. Synaptic models

We consider synaptic models that are quantal, stochastic and exhibit short-term plasticity. The synaptic-dynamics models feature *n* sites where a vesicle can be present for release. If a vesicle is present just before the *m*th pulse then it is released with probability *u*_*m*_. Between the *m*th and (*m* + 1)th pulses an empty vesicle site can be restocked with probability *g*_*m*_. Both release (given a presynaptic AP) and restock events are independent between release sites. The probabilities themselves are deterministic in that they depend on the model parameters only and can be calculated in advance if the times of the action-potentials *t*_*m*_ are known. This formulation encompasses a very broad range of models of short-term plasticity.

When a vesicle is released, the size of the mini PSP it produces in the postsynaptic cell is modeled by a gamma-distributed random variable (see Methods). The mini PSPs induced by different vesicles are assumed to be independently identically distributed. The mean quantal amplitude is μ_*a*_ and the standard deviation is σ_*a*_. In addition there is a normally-distributed background noise with standard deviation σ_*b*_ that is uncorrelated with EPSP amplitude.

For illustrative purposes we focus on a model of synaptic dynamics that features depression and facilitation (Tsodyks and Markram, 1997; Fuhrmann et al., 2002), though other models for which computer code is also provided are described in Appendix A. Activity reduces synaptic efficacy through vesicle depletion; however, the build-up of Ca^2+^ in a presynaptic terminal means that the probability of release given that a vesicle is present *u* is increased by presynaptic activity. Thus, the response to sustained activity can involve larger individual PSPs than the response to isolated spikes. Here, the model has a probability *p*_0_ of release for an isolated pulse; immediately after an isolated presynaptic action potential the release probability increases to *p*_1_. The release probability *u* returns to its initial value *p*_0_ with a timescale τ_*F*_. Empty release sites are restocked on a timescale of τ_*D*_. The model is fully defined in Methods and its parameters are summarized in Table 1.

**Table 1:** **Table of inferred parameters (top) and dynamic variables (bottom) used in the model of synaptic dynamics**.

**Parameter**	**Interpretation**
*n*	Number of statistically independent release sites
τ_*D*_	Timescale of recovery from depression (*s*)
τ_*F*_	Timescale at which facilitation decays (*s*)
*p*_0_	Initial release probability from a single site (given that a vesicle is present)
*p*_1_	Release probability after a single isolated spike
μ_*a*_	Amplitude mean in response to neurotransmitter from one vesicle (*mV*)
σ_*a*_	Amplitude standard deviation in response to neurotransmitter from one vesicle (*mV*)
σ_*b*_	Standard deviation in postsynaptic voltage trace due to background noise (*mV*)
**Variable**	**Interpretation**
*u*	Dynamic release probability
*g*	Probability that an empty release site is restocked

### 3.2. The nature of the computational problem

We now discuss the aim of Bayesian inference and the difficulties correlations cause in calculating the necessary quantities. We consider that the data is in the form of a set of presynaptic action-potential times *t*_1_, *t*_2_, ⋯ , *t*_*M*_ and post-synaptic amplitudes *A*_1_, *A*_2_ ⋯  *A*_*M*_. The aim of the inference procedure is to calculate the probability densities of the parameters of the model θ = {*n, p*_0_, *p*_1_, τ_*D*_⋯ } given the observed presynaptic action potential times {*t*_1_, ⋯ , *t*_*M*_} and postsynaptic amplitudes {*A*_1_, ⋯*A*_*M*_}. Bayesian inference utilizes the fact that the probability of a particular set of parameters being true, given some observed data, is proportional to the probability of observing that data given that those parameters are correct:

(9) $$\begin{array}{l} \text{P}\left(\theta |A_{M},A_{M-1},\cdots \;,A_{1}\right)\propto L\left(A_{M},A_{M-1},\cdots \;,A_{1}|\theta \right). \end{array}$$

The term L on the right-hand side is referred to as the likelihood function. A-priori calculating the likelihood appears computationally infeasible as naively it might be expected to grow exponentially with the number of observed amplitudes *M*. For example, consider a case with *n* possible release sites and a pair (*M* = 2 of presynaptic spikes. Then the likelihood L is given by

(10) $$\begin{array}{l} L\left(A_{2},A_{1}|\theta \right)=\overset{n}{\underset{k_{2}=0}{\sum }}\overset{n}{\underset{k_{1}=0}{\sum }}\text{P}\left[A_{2}|k_{2}\right]\text{P}\left[A_{1}|k_{1}\right]\text{P}\left[k_{2},k_{1}\right] \end{array}$$

where *k*_*m*_ is the number of vesicles released by the *m*th spike. Because of the nested sums there are (*n* + 1)^2^ additive terms in this expansion, and more generally the number of terms in the expansion grows exponentially with the number of presynaptic action potentials ~ (*n* + 1)^*M*^. Written in this form it is clear that the problem becomes computationally prohibitive for long trains of presynaptic spikes and this is what makes calculation of the likelihood difficult for the complete model. The complexity arises from the quantal part of the likelihood P[*k*_2_, *k*_1_]; the individual amplitudes *A*_*m*_ are dependent only on the number of vesicles *k*_*m*_ released by each action potential.

Note that if correlations are ignored and the approximation P(*k*_2_, *k*_1_) ≃ P(*k*_2_)*P*(*k*_1_) made, then the likelihood factorizes and reduces to a product form

(11) $$\begin{array}{l} L\left(A_{2},A_{1}|\theta \right)=\left(\overset{n}{\underset{k_{2}=0}{\sum }}\text{P}\left[A_{2}|k_{2}\right]\text{P}\left[k_{2}\right]\right)\left(\overset{n}{\underset{k_{1}=0}{\sum }}\text{P}\left[A_{1}|k_{1}\right]\text{P}\left[k_{1}\right]\right) \end{array}$$

that is much more computationally tractable in that only 2(*n* + 1) terms are required. This approach was taken by Costa et al. (2013) and combined with an additional approximation that neglected quantal synaptic components to focus on the mean effects of short-term plasticity. For the full probability density in which correlations are retained, it is not possible to factorize the likelihood into a scalar product in this way. However, we will show in the following sections that it is possible to use a Markovian property of this likelihood to factorize the calculation into a matrix product.

### 3.3. Joint probability for serial release events

The quantal component of the likelihood is most problematic; to illustrate the method of tractably calculating the full likelihood we will first consider the joint probability of paired release events P(*k*_2_, *k*_1_). The generalization to a train of many presynaptic action potentials is straightforward. Note that knowing the number of release events at a particular action potential does not specify the state of the system; however, knowing the number of occupied release sites before a spike does fully specify the state of system. This is the Markovian property that makes likelihood calculation possible. We call *y*_*m*_ the number of available vesicles present just before the *m*th action potential. Note that the expected value of *y*_*m*_, $\text{E}\left[y_{m}\right]=n{\langle x\rangle }_{m}^{⊖}$, where ${\langle x\rangle }_{m}^{⊖}$ obeys Equation (2). Using this notation we can write the paired release probability in a more verbose form

(12) $$\begin{array}{l} \text{P}\left(k_{2},k_{1}\right)=\overset{n}{\underset{y_{2}=0}{\sum }}\overset{n}{\underset{y_{1}=0}{\sum }}\text{P}\left(k_{2},y_{2},k_{1},y_{1}\right). \end{array}$$

It is now possible to factorize the probability on the right-hand-side of the above equation. First we use the product rule to expand as follows

(13) $$\begin{array}{l} \text{P}\left(k_{2},y_{2},k_{1},y_{1}\right)=\text{P}\left(k_{2},y_{2},k_{1}|y_{1}\right)\text{P}\left(y_{1}\right) \end{array}$$

(14) $$\begin{array}{l} \;=\text{P}\left(k_{2},y_{2}|k_{1},y_{1}\right)\text{P}\left(k_{1}|y_{1}\right)\text{P}\left(y_{1}\right) \end{array}$$

(15) $$\begin{array}{l} \;=\text{P}\left(k_{2}|y_{2},k_{1},y_{1}\right)\text{P}\left(y_{2}|k_{1},y_{1}\right)\\ \;\text{P}\left(k_{1}|y_{1}\right)\text{P}\left(y_{1}\right) \end{array}$$

(16) $$\begin{array}{l} \;=\text{P}\left(k_{2}|y_{2}\right)\text{P}\left(y_{2}|k_{1},y_{1}\right)\text{P}\left(k_{1}|y_{1}\right)\text{P}\left(y_{1}\right) \end{array}$$

where in the last step we have used the Markovian property of the occupancy variable. Note also that this is an iterative procedure, in which we can factorize the joint probability starting with the first action potential and then the second, that can be continued for joint probabilities that are comprised of an arbitrary number of spikes. For example, for the case of three action potentials it is only necessary to multiply the two-spike case by P(*k*_3_|*y*_3_)P(*y*_3_|*k*_2_, *y*_2_) with the generalization to higher numbers of spike trains obvious. Inserting the final result in Equation (16) of this factorization into Equation (12) results in the following form for the two-spike case

(17) $$\begin{array}{l} \text{P}\left(k_{2},k_{1}\right)=\overset{n}{\underset{y_{2}=0}{\sum }}\overset{n}{\underset{y_{1}=0}{\sum }}\left[\text{P}\left(k_{2}|y_{2}\right)\right]\left[\text{P}\left(y_{2}|k_{1},y_{1}\right)\text{P}\left(k_{1}|y_{1}\right)\right]\left[\text{P}\left(y_{1}\right)\right] \end{array}$$

where the square parentheses have been used to isolate components depending on *k*_2_ or *k*_1_ or neither. This form looks like an inner product and can be written in matrix-vector form (using bra-ket notation) as

(18) $$\begin{array}{l} \text{P}\left(k_{2},k_{1}\right)=\langle l_{2}|q_{1}|r_{0}\rangle \end{array}$$

where 〈*l*_2_| is a row vector dependent on *k*_2_, *q*_1_ is an (*n* + 1) by (*n* + 1) matrix dependent on *k*_1_ and |*r*_0_〉 is a column vector that comprises the initial conditions. Typically P(*y*_1_) = δ_*y*_1, *n*__ so that |*r*_0_〉 has one non-zero entry to indicate that the synapse is initially fully stocked with vesicles. Note also that the case of three action potentials is straightforward

(19) $$\begin{array}{l} \text{P}\left(k_{3},k_{2},k_{1}\right)=\langle l_{3}|q_{2}q_{1}|r_{0}\rangle \end{array}$$

with obvious generalization to higher numbers of spikes. The joint release probability can therefore be reduced to matrix multiplication. The entries of the left row vector and matrices generally comprise two forms. The first form is simply the number of release events *k*_*m*_ chosen from the occupancy *y*_*m*_, using the current probability of release *u*_*m*_ and is therefore binomial

(20) $$\begin{array}{l} \text{P}(k_{m}|y_{m})=\left(\begin{array}{c} y_{m}\\ k_{m} \end{array}\right)u_{m}^{k_{m}}{\left(1-u_{m}\right)}^{y_{m}-k_{m}}. \end{array}$$

The second form gives the occupancy *y*_*m*+1_ given *k*_*m*_ releases from an occupancy *y*_*m*_ at the previous action potential. This implies that there were *n* − *y*_*m*_ + *k*_*m*_ empty release sites just after the *m*th pulse. We require there to be *n* − *y*_*m*+1_ empty sites just before the (*m* + 1)th pulse which means that *y*_*m*+1_ − *y*_*m*_ + *k*_*m*_ sites were restocked. Let *g*_*m*_ be the restock probability of a single empty release site between time *t*_*m*_ and *t*_*m*+1_

(21) $$\begin{array}{l} \text{P}(y_{m+1}|k_{m},y_{m})=\left(\begin{array}{c} n-y_{m}+k_{m}\\ y_{m+1}-y_{m}+k_{m} \end{array}\right)g_{m}^{y_{m+1}-y_{m}+k_{m}}\\ \;{\left(1-g_{m}\right)}^{n-y_{m+1}} \end{array}$$

where this quantity depends on the time between spikes for the synaptic-dynamics model (and all other common synaptic models).

### 3.4. Joint probability for serial EPSP amplitudes

We can now use the factorized form for the serial quantal release events to calculate the full likelihood, which is the joint probability density of seeing amplitudes *A*_1_ and *A*_2_ given the parameter set.

(22) $$\begin{array}{l} L\left(A_{2},A_{1}|\theta \right)=\underset{y_{2}}{\sum }\underset{y_{1}}{\sum }\left[\overset{n}{\underset{k_{2}=0}{\sum }}\text{P}\left[A_{2}|k_{2}\right]\text{P}\left[k_{2}|y_{2}\right]\right]\\ \;[\overset{n}{\underset{k_{1}=0}{\sum }}\text{P}\left[y_{2}|k_{1},y_{1}\right]\text{P}\left[A_{1}|k_{1}\right]\text{P}\left[k_{1}|y_{1}\right]]\\ \;[\text{P}\left(y_{1}\right)]. \end{array}$$

The probabilities P[*A*_1_|*k*_1_] and P[*A*_2_|*k*_2_] for the observed amplitudes given that a certain number of vesicles were released are defined by Equation (8). The form of Equation (22) can again be interpreted as an inner product which can be written in bra-ket notation

(23) $$\begin{array}{l} L\left(A_{2},A_{1}|\theta \right)=\langle L_{2}|Q_{1}|R_{0}\rangle \end{array}$$

where 〈*L*_2_| is a row vector dependent on *A*_2_, *Q*_1_ is a matrix dependent on *A*_1_ and |*R*_0_〉 is a column vector with the initial configuration before the first action potential. This quantity is relatively straightforward to compute and, importantly, does not grow exponentially in computational complexity for higher numbers of action potentials. For example, for three spikes we have

(24) $$\begin{array}{l} L\left(A_{3},A_{2},A_{1}|\theta \right)=\langle L_{3}|Q_{2}Q_{1}|R_{0}\rangle \end{array}$$

with the generalization to higher numbers of presynaptic spikes straightforward.

### 3.5. Inferring quantal parameters from synthetic data

**Figure 1:**
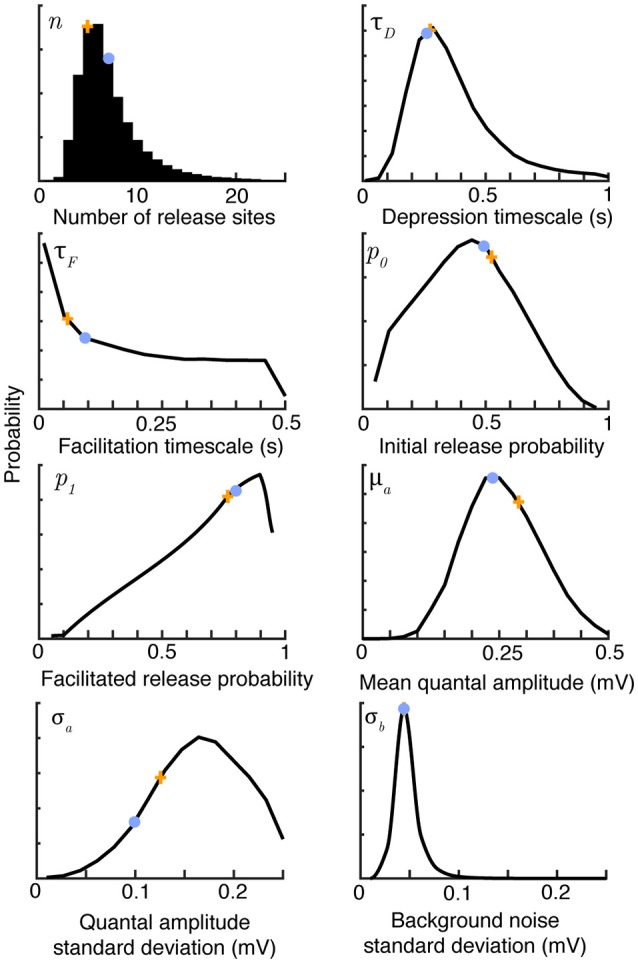
**Bayesian inference provides parameter distributions from five sweeps of synthetic data comprising 30 regular spikes at 30 Hz**. Marginal posterior distributions (black), maximum *a-posteriori* estimates (orange crosses) and true parameter values (light blue dots) for the parameters of the synaptic model summarized in Table 1. Posteriors shown after 10^6^ Metropolis-Hastings samples. The true values were *n* = 7, τ_*D*_ = 0.25 s, τ_*F*_ = 0.2 s, *p*_0_ = 0.6, *p*_1_ = 0.8, μ_*a*_ = 0.25 mV, σ_*a*_ = 0.1 mV and σ_*b*_ = 0.05 mV.

The methodology just described is first applied to synthetic data to test how well the correlated likelihood function can recover quantal and dynamic parameters (Figure 1). Here the synaptic-dynamics model is used to generate sweeps of synthetic amplitude trains. For this model, the eight parameters to infer are the release site number *n*, initial release probability *p*_0_, facilitated release probability after an isolated spike *p*_1_, depression timescale τ_*D*_, facilitation timescale τ_*F*_, mean quantal amplitude μ_*a*_, standard deviation in quantal amplitude σ_*a*_, and standard deviation of background noise σ_*b*_.

Figure 1 shows marginal posterior distributions of these eight parameters given five simulated sweeps, each of 30 regular spikes at 30Hz. The posterior distributions reflect the true parameters well for all synaptic parameters with the exception of the facilitation timescale τ_*F*_ and quantal amplitude standard deviation σ_*a*_. These parameters have been observed to be hard to estimate in previous studies, with Costa et al. (2013) finding broad distributions for τ_*F*_, and Bhumbra and Beato (2013) and Barri et al. (2016) noting similar uncertainties in their estimates of quantal variability. The correlated Bayesian method does not qualitatively change these results, but makes the best use of available data to accurately estimate the uncertainty. The posterior distributions narrow with more data, but it is also possible to change experimental protocols to improve estimates. Costa et al. (2013) note that when the stimulation process is Poisson, rather than periodic, estimates of the time constants τ_*D*_ and τ_*F*_ using their method are improved due to the broader range of interspike intervals. This is equally true of the correlated Bayesian method. Estimates of σ_*a*_ could be improved by a very high stimulation rate that typically causes either 0 or 1 vesicles to release with each spike. Note that with typical delays between sweeps of 15 s, collecting this dataset required just over a minute of experimental time, giving a relatively sparse dataset that nevertheless still allows good estimates of the underlying synaptic parameters.

**Figure 2:**
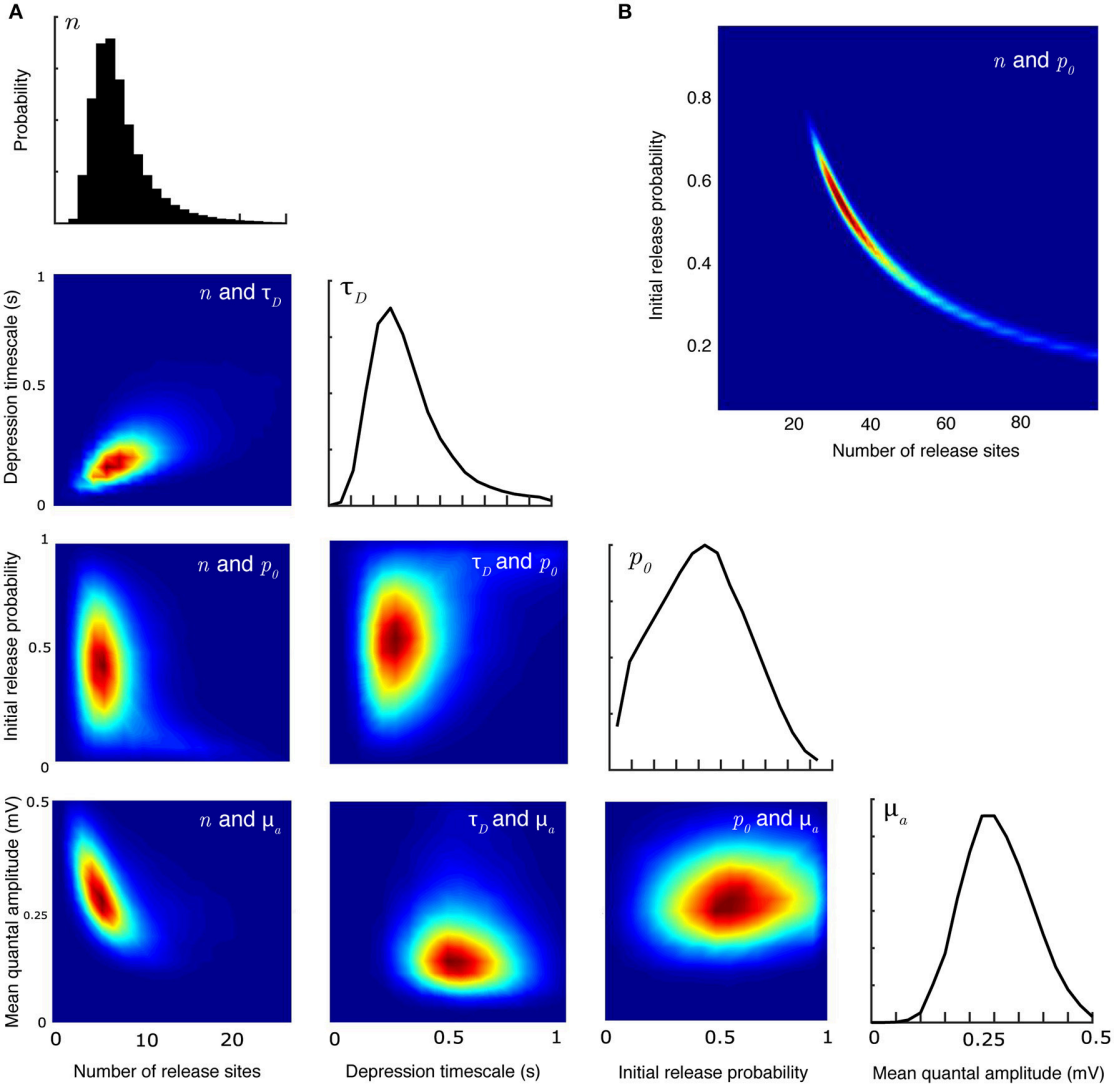
**Joint parameter estimates for the synaptic-dynamics model. (A)** Pairwise and individual posterior marginals for release-site number *n*, depression timescale τ_*D*_, initial release probability *p*_0_, and mean quantal amplitude μ_*a*_. True parameter values and data are the same as Figure 1. Colorbars for the values of the posterior distributions are not shown; the relative differences in value show the shape and sharpness of the pairwise posteriors for each pair of parameters. **(B)** Pairwise posterior marginal for release site number *n* and initial release probability *p*_0_ for a case where the true values were *n* = 35 and *p*_0_ = 0.50 showing a strong anticorrelation. All posteriors shown after 10^6^ Metropolis-Hastings samples.

A major advantage of the Bayesian method over a maximum likelihood approach is that it can recover the full distribution of parameters. This allows determination of the covariances between different parameters. Figure 2 plots the joint posterior distributions of certain pairs of parameters (in total there are 28 possible pairs for the synaptic-dynamics model considered here). Figure 2A shows the relationship between release site number *n*, depression timescale τ_*D*_, initial release probability *p*_0_, and mean quantal amplitude μ_*a*_. The inverse relationship between estimates of *n* and μ_*a*_ can be anticipated beause the mean EPSP size will always depend on the product of these two quantities. Note in particular that the relationship between release probability and both *n* and τ_*D*_ has a characteristic curved shape that is not apparent from looking at the individual marginal distributions. This is even more apparent (Figure 2B) for larger values of *n* that can be seen in some central synapses (Loebel et al., 2009, 2013).

### 3.6. Experiment: changing synaptic dynamics under adenosine application

**Figure 3:**
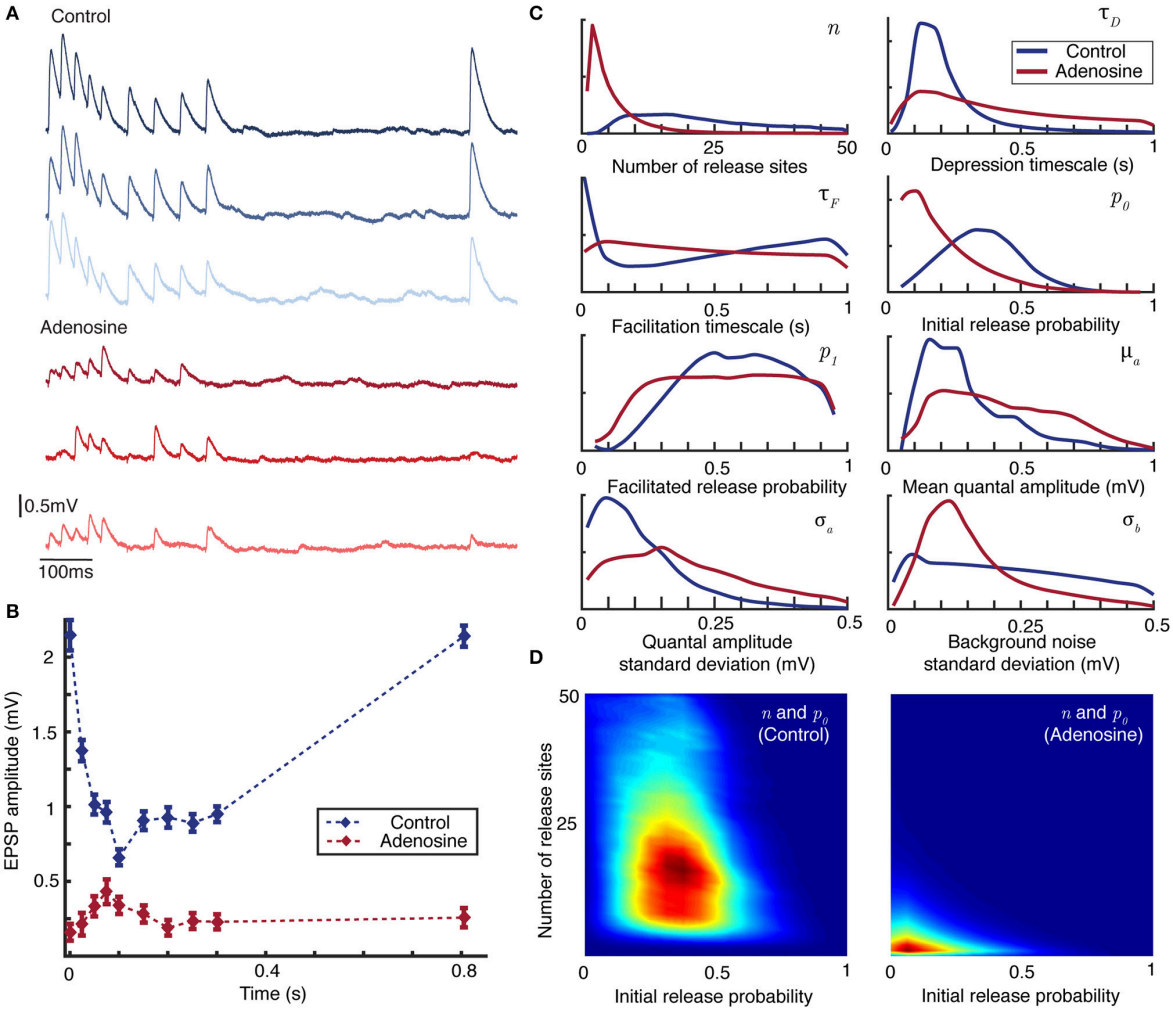
**Bayesian inference captures the shift in synaptic dynamics under application of adenosine. (A)** Individual postsynaptic voltage traces under control (top) and adenosine (bottom) conditions. **(B)** Mean EPSP size for each spike in the stimulation protocol under control (blue) and adenosine (red) conditions. Bars show standard error. **(C)** Marginal posterior distributions for the parameters of the synaptic model in the control (blue) and adenosine (red) conditions. **(D)** Pairwise posterior marginals for number of active release sites *n* and initial release probability *p*_0_ before (left) and after (right) application of adenosine. Posteriors shown after 5 × 10^6^ Metropolis-Hastings samples.

The neuromodulator adenosine is implicated (Kerr et al., 2013) in the developmental shift from dominant depression at juvenile synapses to weak facilitation at mature synapses (Reyes and Sakmann, 1999). Adenosine acts via *A*1 receptors to ultimately reduce the probability of vesicle release (Dunwiddie and Fredholm, 1989). Measurement of synaptic dynamics under control conditions and then during bath-application of adenosine therefore provides a convenient experimental protocol to test the inference method. For the control case an initially depressing juvenile connection was stimulated 40 times with nine periodic presynaptic spikes at 40 and 20 Hz (see Figure 3A) followed by a recovery spike, with the postsynaptic response recorded. Adenosine (100 μM) was then bath-applied to the slice (see Methods) and the stimulus protocol repeated.

Figure 3A plots individual postsynaptic voltage traces before and after the application of adenosine; Figure 3B shows the change in average EPSP size. The marginal maximum-likelihood estimates for the depression timescale τ_*D*_ and mean quantal amplitude μ_*a*_ are similar between the control and adenosine datasets (Figure 3C). However, the suppressive effect of adenosine on synaptic transmission is clearly visible in the effective number of release sites *n* and the initial release probability *p*_0_ that drives the shift from predominantly depressing to weakly facilitating synapses. It is also possible to examine the changes in covariance between pairs of parameters inferred from the experimental data (Figure 3D). Considering active release sites *n* and initial release probability *p*_0_ together makes particularly apparent the shift in synaptic transmission.

### 3.7. Comparison with methods that neglect serial correlations

**Figure 4:**
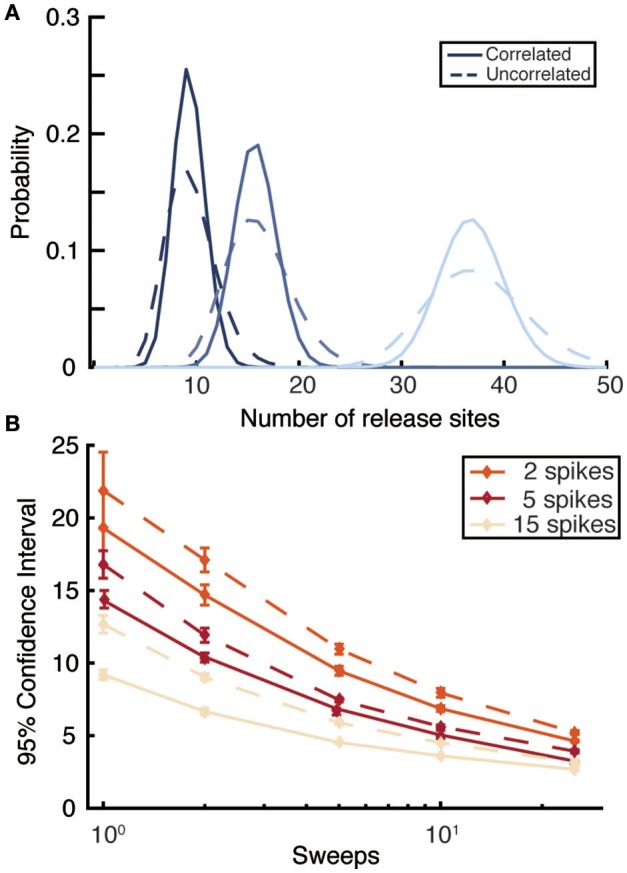
**Comparison of likelihood functions that do or do not account for serial correlations in synaptic amplitudes. (A)** Posterior distributions for release site number *n* computed by correlated (solid) and uncorrelated (dashed) likelihood functions for three different values of *n* (*n* = 8 dark black; *n* = 15 middle blue; and *n* = 35 light blue) for a single sweep of 5 spikes regularly distributed at 30Hz. **(B)** 95% confidence intervals for correlated (solid) and uncorrelated (dashed) likelihood functions as a function of the number of sweeps for different numbers of spikes per train. Spikes occur at 30Hz, the true value of *n* is 35, and averages are taken over 10 realizations. Other parameters are the same as for Figure 1 (light-blue dots).

Previous Bayesian inference methods have demonstrated that an uncorrelated likelihood function can accurately infer the quantal (Bhumbra and Beato, 2013) and mean dynamic (Costa et al., 2013) parameters of a synapse. It can therefore be asked under what conditions does the exact likelihood function, which accounts for correlations, provide an improvement over existing methods. Synapses with low numbers of release sites *n*, high release probabilities *u*, or long depression timescales τ_*D*_ have the strongest correlations between EPSPs. High release probabilities *u* can arise either at strongly depressing synapses, with a high value of *p*_0_, or facilitating synapses where the stimulation protocol causes large values of *u*(*t*) to arise. In addition to these, at least partly, physiological factors, the correlated likelihood function is superior in conditions of sparse data. When only a few PSPs are available per sweep or, more importantly, only a few sweeps are available correlations within a spike train are relatively more important. To quantify this, we compared the full likelihood function described above with an approximated likelihood calculated by ignoring correlations (calculated using forms like Equation 11). The approximate likelihood did not account for the observed previous PSP amplitudes within a sweep, only their distribution of probabilities given by the model parameters and previous spike times. As expected, the uncorrelated likelihood function gave broader posterior distributions (Figure 4A) with this effect diminishing as more data is added, either in the form of more EPSPs per sweep or more independent sweeps (Figure 4B). Overall, the exact likelihood function that accounts for correlations provides superior inference on synaptic parameters. It is possible to obtain accurate constraints on synaptic parameters with only a few sweeps, meaning that experiments could capture a snapshot of synaptic properties in a small time window during protocols that change synaptic properties on timescales of tens of seconds rather than tens of minutes.

## 4. Discussion

We have presented a method for exactly and efficiently calculating the probability of a given train of PSP amplitudes for dynamical synapses with the utility and robustness of the method demonstrated on synthetic and experimental data. This method, presented earlier in Bird (2016) is equivalent to that simultaneously and independently discovered by Barri et al. (2016) in their expectation-maximization approach, and represents a combination and extension of the recent work of Bhumbra and Beato (2013) on the exact likehood of isolated events and Costa et al. (2013) on the approximated likelihood of serial events. By considering quantal and dynamic properties together, the method described accounts for information that is necessarily neglected when each component is examined in isolation. The advance renders the calculation of the likelihood required for Bayesian inference practical for a variety of models of short-term synaptic plasticity. Moreover, unlike approaches that have relied on mean-variance analysis, it is applicable to single-sweep experiments and so is suitable for *in-vivo* scenarios where presynaptic firing is uncontrolled, but can be monitored.

The likelihood calculation that makes this inference possible is flexible and can be extended to a number of common synaptic models, allowing for examination of augmented recovery (Wang and Kaczmarek, 1998; Hosoi et al., 2007), release-independent depression with frequency-dependent recovery (Fuhrmann et al., 2004), and receptor desensitization (Jones and Westbrook, 1996; Otis et al., 1996). Four such models are described in Appendix A with associated computer code in the MATLAB and JULIA environments to be found in the Supplementary Material. Another natural and straightforward extension of the methodology presented here is to not assume that all sites are initially occupied but have the initial state of the system as a parameter to be inferred. This scenario is relevant for *in-vivo* experiments where there is no natural break in the presynaptic activity: in this case the release site occupancy and state of the dynamic release probability would be unknown.

## Author contributions

AB and MR wrote the paper, AB and MR derived the equations, AB made the figures, AB wrote the MATLAB code and MR wrote the JULIA code, MW supervised experimental data collection.

## Funding

We acknowledge funding through a Warwick Systems Biology Doctoral Training Centre fellowship to AB, funded by the UK BBSRC funding agency (BBSRC Grant No. BB/G530233/1), and funding to MR under BBSRC Grant No. BB/J015369/1.

### Conflict of interest statement

The authors declare that the research was conducted in the absence of any commercial or financial relationships that could be construed as a potential conflict of interest.

## Supplementary material

Supplementary Material comprises MATLAB and JULIA code to run Bayesian inference using the depression (DEP), depression and facilitation (FAD), release-independent depression (RID) and release-independent depression with frequency-dependent recovery (FDR) synaptic models described, in the Methods and Appendix A.

## Appendix A

### Extension to other synaptic models

The likelihood calculation that was illustrated in the main text for a model with depression and facilitation can be straightforwardly adapted to other commonly used models of synaptic dynamics. These comprise models in which the restock probability *g*_*m*_ between presynaptic action potentials *m* and *m* + 1 and probability of release at the arrival of the *m*th action potential *u*_*m*_ depends only on the pattern of presynaptic activity. As part of the Supplementary Material we provide computer code for four such models, which are now described below with the synaptic parameters and dynamic variables tabulated in Table A1.

**Table A1:** **Extended table of inferred parameters (top) and dynamic variables (bottom) used in the synaptic models discussed in Appendix A**.

**Parameter**	**Interpretation**
*n*	Number of statistically independent release sites
τ_*D*_	Timescale of recovery from depression (s)
τ_*F*_	Timescale at which facilitation decays (s)
*p*_0_	Initial release probability from a single site (given that a vesicle is present)
*p*_1_	Release probability after a single isolated spike
μ_*a*_	Mean voltage response to neurotransmitter from a single vesicle (mV)
σ_*a*_	Standard deviation of voltage response distribution to neurotransmitter contained in a single vesicle (mV)
σ_*b*_	Standard deviation in postsynaptic voltage trace due to background noise (mV)
τ_*I*_0__	Initial recovery timescale from RID (s)
τ_*I*_1__	Recovery timescale from RID after a single isolated pulse (s)
ς_*I*_	Decay timescale of FDR (s)
*u*	Dynamic release probability
*g*	Probability that an empty release site is restocked
τ_*I*_	Dynamic RID recovery timescale (s)

#### (i) Depression only - DEP model

This is perhaps the simplest model of short-term synaptic plasticity and features only vesicle depletion and restock (Tsodyks and Markram, 1997; Fuhrmann et al., 2002). The occupation of a single release site is governed by the stochastic differential Equation (1). The mean-occupancy recursion relations for the model are given by Equations (2) and (3) with a constant release probability *u*_*m*_ = *p*_0_. The Poissonian restock of empty release sites occurs at a constant rate 1/τ_*D*_ and so in this case the restock probability *g*_*m*_ is given by Equation (4).

#### (ii) Depression and facilitation - DAF model

This is the model described in the main text (Varela et al., 1997; Tsodyks et al., 1998; Fuhrmann et al., 2002) and applies to facilitating synapses. The probability of restock is defined by Equation (4) and the probability of release *u*_*m*_ is given by recursion Equation (6).

#### (iii) Release-independent depression - RID model

This model was introduced (Fuhrmann et al., 2004) for synapses that do not display facilitation and considers a different form of depression which is uncorrelated with the preceding EPSP amplitudes. Release-independent depression is a reduction in release probability *u*_*m*_ caused by spiking activity which decays on a timescale τ_*I*_0__ (it can be thought of as a kind of anti-facilitation). The release probability immediately after an isolated pulse is again called *p*_1_ but in contrast to facilitation *p*_1_ < *p*_0_. In this formalism the release probability *u*(*t*) obeys

(A1) $$\begin{array}{l} \frac{du}{dt}=\frac{p_{0}-u}{\tau_{I_{0}}}-\frac{u}{p_{0}}\left(p_{0}-p_{1}\right)\underset{t_{m}}{\sum }\delta \left(t-t_{m}\right) \end{array}$$

where *t*_*m*_ are the times of the presynaptic action-potentials. The values of *u*(*t*) just after the *m*th and before the (*m* + 1)th action potentials ($u_{m}^{⊕}$ and *u*_*m*+1_ respectively) are defined by the following recursion relations

(A2) $$\begin{array}{l} \;u_{m}^{⊕}=u_{m}-u_{m}\left(\frac{p_{0}-p_{1}}{p_{0}}\right)\text{and}\\ u_{m+1}=p_{0}+\left(u_{m}^{⊕}-p_{0}\right)e^{-T_{m}/\tau_{I_{0}}}. \end{array}$$

The restock probability *g*_*m*_ is given by Equation (4) and is common to the previous two models.

#### (iv) Release-independent depression with frequency dependent recovery - FDR model

The recovery from release-independent depression is often seen to be frequency dependent (Fuhrmann et al., 2004). To account for this the timescale τ_*I*_(*t*) is now a dynamic variable with initial value τ_*I*_0__, magnitude after an isolated spike of τ_*I*_1__ and decay timescale ς_*I*_. The relevant equations are now

(A3) $$\begin{array}{l} \frac{du}{dt}=\frac{p_{0}-u}{\tau_{I}}-\frac{u}{p_{0}}\left(p_{0}-p_{1}\right)\underset{t_{m}}{\sum }\delta \left(t-t_{m}\right) \end{array}$$

(A4) $$\begin{array}{l} \frac{d\tau_{I}}{dt}=\frac{\tau_{I_{0}}-\tau_{I}}{\varsigma_{I}}-\frac{\tau_{I}}{\tau_{I_{0}}}\left(\tau_{I_{0}}-\tau_{I_{1}}\right)\underset{t_{m}}{\sum }\delta \left(t-t_{m}\right) \end{array}$$

The dynamic *RID* recovery timescale τ_*I*_(*t*) obeys Equation (A4). The values of τ_*I*_(*t*) just after the *m*th and before the (*m* + 1)th action potentials ($\tau_{Im}^{⊕}$ and $\tau_{Im+1}^{⊖}$ respectively) are defined by the following recursion relations

(A5) $$\begin{array}{l} \;\tau_{Im}^{⊕}=\tau_{Im}^{⊖}-\tau_{Im}^{⊖}\left(\frac{\tau_{I_{0}}-\tau_{I_{1}}}{\tau_{I_{0}}}\right)\text{and}\\ \;\tau_{Im+1}^{⊖}=\tau_{I_{0}}+\left(\tau_{Im}^{⊕}-\tau_{I_{0}}\right)e^{-T_{m}/\varsigma_{I}}. \end{array}$$

The release probability *u*(*t*) obeys Equation (A3) and can also be defined recursively, with *u*_*m*+1_ the release probability at the (*m* + 1)th spike at time *t*_*m*+1_ given by

(A6) $$\begin{array}{l} u_{m+1}=p_{0}+\frac{(u_{m}^{⊕}-p_{0}){\left(\tau_{Im}^{⊕}\right)}^{\frac{\zeta_{I}}{\tau_{I_{0}}}}}{{\left[\tau_{Im}^{⊕}-\tau_{I_{0}}\left(1-e^{\frac{T_{m}}{\varsigma_{I}}}\right)\right]}^{\frac{\zeta_{I}}{\tau_{I_{0}}}}} \end{array}$$

where $u_{m}^{⊕}=u_{m}-u_{m}\left(\frac{p_{0}-p_{1}}{p_{0}}\right)$ is the release probability immediately following the *m*th spike.

## Appendix B

### The likelihood convolution integral

The convolution integral for the amplitude distribution Equation (8) is computed a large number of times. There are two difficulties in doing this efficiently: (i) evaluating a gamma function for large shape parameters and (ii) finding reasonable bounds for the range of integration.

#### Evaluating gamma functions

When μ_*a*_ ≪ σ_*a*_ the argument of the gamma function in the denominator of Equation (8) can grow very large in order to normalize the distribution. To avoid issues with this, we note that Stirling's approximation allows evaluation of the gamma function with large arguments

$$\begin{array}{l} \Gamma \left(n+1\right)\\ \;\approx \sqrt{2\pi n}{\left(\frac{n}{e}\right)}^{n}\left(1+\frac{1}{12n}+\frac{1}{288n^{2}}-\frac{139}{51840n^{3}}-\frac{571}{2488320n^{4}}\right) \end{array}$$

and introduce a variable κ(*n*) such that

$$\begin{array}{l} \kappa \left(n\right)=\frac{1}{\Gamma \left(n+1\right)}\sqrt{2\pi n}{\left(\frac{n}{e}\right)}^{n}\\ \;\approx {\left(1+\frac{1}{12n}+\frac{1}{288n^{2}}-\frac{139}{51840n^{3}}-\frac{571}{2488320n^{4}}\right)}^{-1}. \end{array}$$

For small values of *n* the function κ(*n*) can be evaluated exactly, whereas for larger arguments the second, approximate form is used.

#### Bounding the range of integration

The second difficulty involves finding bounds for the range of integration in Equation (8). Introducing γ = β − 1 and *y*_*_ = γ/λ, and substituting κ(γ) for the gamma function, Equation (8) can be rewritten as

$$\begin{array}{l} \text{P}\left[A|k\right]=\frac{\kappa \left(\gamma \right)}{2\pi \sigma_{b}}\frac{1}{\sqrt{y_{*}/\lambda }}\int_{0}^{\infty }dy\\ \;\exp\left(-\lambda \left(y-y_{*}\right)+\gamma \log\left(y/y_{*}\right)-\frac{{\left(A-y\right)}^{2}}{2\sigma_{b}^{2}}\right) \end{array}$$

which is in a form that is straightforward to integrate numerically, once reasonable integration bounds are identified. To this end we note that by completing the squares in the exponential and introducing *z* = *y*/σ_*b*_ the integrand (ignoring constant prefactors) can be written as

$$\begin{array}{l} f\left(z\right)=z^{\gamma }e^{-{\left(z-2t\right)}^{2}/2} \end{array}$$

where *t* = (*A*/σ_*b*_ − λσ_*b*_)/2. The integrand has a single positive maximum $z_{*}=t+\sqrt{t^{2}+\gamma }$ (note that γ = β − 1 > 0 because the assumption is made that μ_*a*_ > σ_*a*_, see Methods). Our method then is to perform the *y* integral over an equivalent range of *z* for which $f\left(z\right)>f\left(z_{*}\right)e^{-x}$ where *x* is some sufficiently large power (typically in the range 10–15) with the boundaries of the *z* range efficiently found using a Newton-Raphson method.
