# Supplementary material for: Bayesian Inference of Synaptic Quantal Parameters from Correlated Vesicle Release
Source: Front Comput Neurosci. 2016 Nov 25;10:116. doi: 10.3389/fncom.2016.00116 (PMC5122579; doi:10.3389/fncom.2016.00116)
Supplement: Supplementary file 1 [file Presentation1.ZIP › MATLAB (all models)/DEP (depression)/Overview.rtf]

%%%%%%%%%%%%%%%%%%%%%%%%%%%%%%%%%%%%%%%%%%%%%%%%%%%%%%%%%%%%%%%%%%%%%%%%%%%%%%%%%%%%%%%%%%%%%%%%%%%%%%%%%%%%%%%%%%%%%%%%%%%%%%%%%%%%%%%%%%%%%%%%%%%%%%%%%%%%%%%%%%%% COPYRIGHT: Alexander Bird (2016).% This code is free software: you can redistribute it and/or modify% it under the terms of the GNU General Public License as published by% the Free Software Foundation, either version 3 of the License, or% (at your option) any later version.%% This program is distributed in the hope that it will be useful,% but WITHOUT ANY WARRANTY; without even the implied warranty of% MERCHANTABILITY or FITNESS FOR A PARTICULAR PURPOSE.  See the% GNU General Public License for more details.%% See <http://www.gnu.org/licenses/> for a copy of  % the GNU General Public License%%%%%%%%%%%%%%%%%%%%%%%%%%%%%%%%%%%%%%%%%%%%%%%%%%%%%%%%%%%%%%%%%%%%%%%%%%%%%%%%%%%%%%%%%%%%%%%%%%%%%%%%%%%%%%%%%%%%%%%%%%%%%%%%%%%%%%%%%%%%%%%%%%%%%%%%%%%%%%%%%%%Guide to using the Bayesian inference function.This file contains four functions and one script. The script ‘Example’ generates synthetic data and runs a Bayesian inference MCMC on it to recover the posterior marginals, before plotting these. It provides examples of the usage of the four other functions. In brief, they have the following functions:‘Bayes’ - Runs Bayesian inference MCMC on input data.‘Likelihood’ - Internal function, determines the likelihood of a dataset given a set of model parameters.‘PosteriorPlot’ - Plots posterior marginals.’SyntheticData’ - Generates synthetic dataset to test inference.
